# Investigation of Homocysteine-Pathway-Related Variants in Essential Hypertension

**DOI:** 10.1155/2012/190923

**Published:** 2012-10-23

**Authors:** Javed Y. Fowdar, Marta V. Lason, Attila L. Szvetko, Rodney A. Lea, Lyn R. Griffiths

**Affiliations:** Genomics Research Centre, Griffith Health Institute, Gold Coast Campus, Griffith University, Southport, QLD 4222, Australia

## Abstract

Hyperhomocysteinemia (hHcy) has been associated with an increased risk of cardiovascular disease and stroke. Essential hypertension (EH), a polygenic condition, has also been associated with increased risk of cardiovascular related disorders. To investigate the role of the homocysteine (Hcy) metabolism pathway in hypertension we conducted a case-control association study of Hcy pathway gene variants in a cohort of Caucasian hypertensives and age- and sex-matched normotensives. We genotyped two polymorphisms in the methylenetetrahydrofolate reductase gene (MTHFR C677T and MTHFR A1298C), one polymorphism in the methionine synthase reductase gene (MTRR A66G), and one polymorphism in the methylenetetrahydrofolate dehydrogenase 1 gene (MTHFD1 G1958A) and assessed their association with hypertension using chi-square analysis. We also performed a multifactor dimensionality reduction (MDR) analysis to investigate any potential epistatic interactions among the four polymorphisms and EH. None of the four polymorphisms was significantly associated with EH and although we found a moderate synergistic interaction between MTHFR A1298C and MTRR A66G, the association of the interaction model with EH was not statistically significant (*P* = 0.2367). Our findings therefore suggest no individual or interactive association between four prominent Hcy pathway markers and EH.

## 1. Introduction

Hypertension is defined as a sustained systolic blood pressure of greater than 140 mmHg, a diastolic blood pressure of greater than 90 mmHg, or both [1]. Ninety five percent of hypertensives suffer from essential hypertension (EH) with the remaining 5% exhibiting high blood pressure due to some underlying disorder such as Liddle's syndrome, glucocorticoid-remediable aldosteronism, or apparent mineralocorticoid excess syndrome [1]. Worldwide, about one billion people suffer from hypertension while in Australia at least 30% of men and 20% of women are hypertensive [2]. In addition to the direct costs of treating EH, it is also a risk factor for many cardiovascular diseases (CVD), with EH implicated in 7.5 million deaths annually from ischaemic heart disease and stroke [3]. Determining the risk factors for EH is therefore important for understanding both EH and CVD and may help to develop new treatment or prevention strategies.

There are a number of environmental and clinical risk factors associated with EH including, but not limited to, dietary intake of sodium, alcohol intake, lack of exercise, poor diet, obesity, insulin resistant diabetes, and hyperlipidemia. Although these factors explain a substantial proportion of hypertension susceptibility, it is estimated that up to 60% of the variation in hypertension risk is due to an individual's genetic makeup [4]. Thus, many studies have investigated the genetic component of hypertension using the well-known animal model, the spontaneous hypertensive rat [5], or undertaking genetic association and linkage studies [6] in hypertensive case-control and family cohorts. Investigations into the genetic component of hypertension have mainly focussed on the renin-angiotensin-aldosterone (RAA) system because of its importance in regulating normal blood pressure [7]. Other genes, such as those involved in the central nervous system, vascular-endothelial system, and metabolic system, have also been extensively studied [7].

**Figure 1:**
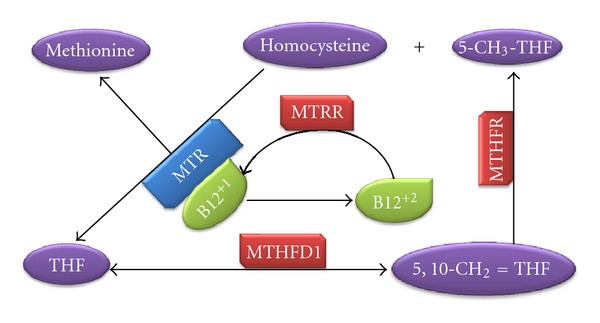
Simplified homocysteine pathway.

The homocysteine (Hcy) pathway has emerged as a strong candidate for EH and many studies have investigated genetic variation underlying hyperhomocysteinemia (hHcy). However, results have so far been inconclusive, with some studies reporting a significant association [8–10] while others have reported no association [11, 12]. The third National Health and Nutrition Examination Survey (NHANES III) reported that people with the highest level of Hcy carried a 2 to 3 fold increase in hypertension prevalence than those with the lowest Hcy level [13]. It is thought that Hcy levels are mainly increased by environmental factors such as lack of folate, vitamin B12, and vitamin B6 in the diet [14]; however, alterations in the Hcy pathway have also been shown to lead to mild hHcy in humans [15]. The Hcy pathway involves the conversion of Hcy to methionine. Briefly, tetrahydrofolate, a folic acid derivative, is converted to 5,10-methylenetetrahydrofolate (5,10-MTHF) by the enzyme methylenetetrahydrofolate dehydrogenase 1 (MTHFD1). 5,10-MTHF is converted to 5-methyltetrahydrofolate by methylenetetrahydrofolate reductase (MTHFR). Ultimately this substrate reacts with Hcy to form methionine and regenerates tetrahydrofolate. Methionine synthase (MTR), in the presence of cobalamin (vitamin B12), regulates this reaction. However over time, cobalamin which is a strong reductant becomes oxidised, thereby inactivating the MTR enzyme. The enzyme methionine synthase reductase (MTRR) reactivates MTR by reducing cobalamin to its original state [16]. A simplified pathway is shown in Figure 1.

One of the most studied genetic variants contributing to hHcy is the C to T single nucleotide polymorphism (SNP) at codon 677 of the MTHFR gene. The C to T substitution causes alanine to be substituted by valine. The TT variant codes for a thermolabile enzyme which has a 50% reduced activity compared to the CC variant [17]. Another SNP in the same gene occurs at codon 1298 with an A to C substitution. This leads to glutamine being substituted by alanine. Although the CC variant also reduces enzymatic activity, with its effect not as drastic as the TT variant occurring at codon 677 [18], both polymorphisms result in a decrease in MTHFR enzyme activity, which decreases production of 5-methyltetrahydrofolate, the necessary substrate for Hcy conversion to methionine. By decreasing levels of 5-methyltetrahydrofolate, these polymorphisms could therefore result in accumulation of Hcy, leading to hHcy [15].

Although the MTRR enzyme does not directly participate in the conversion of Hcy to methionine, the fact that it keeps the MTR enzyme active makes it a key enzyme in Hcy metabolism. A common SNP in MTRR is the A to G substitution at codon 66. This substitution causes isoleucine to be substituted by methionine in the enzyme. It has been reported that the mutant enzyme exhibits a four-fold lower activity in reactivating MTR than the wild type enzyme [19]. This polymorphism has also been associated with increased Hcy levels [20]. The MTHFD1 gene codes for a tri-functional enzyme: 5,10-MTHF dehydrogenase, 5,10-MTHF cyclohydrolase, and 10-formyltetrahydrofolate synthetase. The G1958A polymorphism results in the replacement of arginine by glycine within the synthetase active domain and reduces the enzymatic activity of MTHFD1 by about 26% [16], thereby disrupting methionine synthesis and possibly resulting in increased levels of Hcy.

This study investigated whether there is an association between EH and the MTHFR C677T, MTHFR A1298C, MTRR A66G, and MTHFD1 G1958A variants in an Australian case-control cohort. An interaction analysis using the multifactor dimensionality reduction (MDR) method was also performed to investigate whether specific combinations of genotypes across all four loci contribute to disease status.

MDR analysis is a data mining method used to detect and classify combinations of independent variables such as genotypes or environmental factors that may interact to cause disease. MDR classifies the genotype combinations of two loci (multilocus genotype) into either belonging to a low-risk group or a high-risk group. For example, all possible genotypes at locus 1 (AA, Aa, aa) are paired with each other possible genotype at locus 2 (BB, Bb, bb), giving nine possible multilocus genotypes (AA/BB, AA/Bb, AA/bb, and so on). Each multilocus genotype is then evaluated for the number of cases versus controls, and assigned to be high-risk if the number of cases exceeds the number of controls, corresponding to a ratio >1 for matched populations [21, 22]. If the ratio is <1, the multilocus genotype is defined as low-risk. When numbers are equal, multilocus genotypes can be assigned as affected (high risk), unaffected (low risk), or unassigned. This redefinition of two-dimensional (two-locus) data as one dimension (risk value) is how MDR reduces the complexity of multidimensional data. The risk value dimension can then be analysed to predict the outcome variable (case or control status) using a non-parametric method which is better suited to deal with modelling of high-order interactions in small sample sizes. Non-parametric methods such as MDR are being increasingly used for genetic interaction analysis as they are model-free and are considered more robust than parametric methods [22].

## 2. Methods

### 2.1. Study Population

The study protocol was approved by the Griffith University's Ethics Committee. The study population was composed of 409 hypertensives and 409 age- (±5 years), sex-, and ethnicity-matched normotensive controls, who resided in the South East Queensland region of Australia. All participants were of Caucasian origin. Cases were defined as individuals who were clinically diagnosed as suffering from hypertension and who were taking antihypertensive drugs. Controls were defined as participants who were not taking antihypertensive drugs, and whose blood pressure was less than 140/90 mmHg. Individuals suffering from renal disorders (polycystic kidneys, renovascular disease, parenchymal renal disease), primary aldosteronism, Cushing syndrome, and hypothyroidism were excluded from the study. None of the participants included in the study reported any previous cardiovascular events such as heart attacks or stroke. 53.3% of the population were female and 46.7% were male. The average age of the case group was 63.1 ± 10.9 years and the average age of the control group was 61.0 ± 10.5 years. Peripheral blood samples as well as questionnaires detailing medical history, including blood pressure and prescribed medications, were obtained from all participants. All participants signed informed consent agreements prior to collection of blood and clinical information.

### 2.2. Genotyping Methods

DNA was extracted from blood samples using a modified version of the salting-out method [23]. Two polymorphisms in MTHFR and one polymorphism in MTRR and MTHFD1 were genotyped for all cases and controls. Detailed information regarding polymorphisms and a summary of assay conditions and primer sequences for each polymorphism are listed in Table 1. All PCR buffers, MgCl_2_, GoTaq polymerase were from Promega Corp., Madison, WI, USA; dNTPs, restriction enzymes, and enzyme buffers were from New England Biolabs, Ipswich, MA, USA; SYTO9 dye was from Invitrogen, Carlsbad, CA, USA. Protocol and assays for each polymorphism are described in detail below.

### 2.3. MTHFR Genotyping

The MTHFR C677T polymorphism was genotyped by polymerase chain reaction (PCR) followed by restriction fragment length polymorphism (RFLP) analysis. The PCR protocol was as follows: 1X PCR buffer, 1.75 mM MgCl_2_, 0.2 mM dNTPs, 0.2 uM forward primer, 0.2 uM reverse primer, 1U GoTaq, and 40 ng of DNA. The primer sequences were designed by Frosst [24] and were validated as described in a previous study [25]. The PCR thermocycling conditions were as follows: 95°C for 3 mins, then 94°C for 40 seconds, 69°C for 40 seconds, and 72°C for 1 minute for 35 cycles, followed by a final extension step of 72°C for 5 minutes. The 198 bp PCR products were electrophoresed on a 15 cm 2% agarose gel containing 0.006% ethidium bromide) for 30 mins at 90 V, and then visualised under ultraviolet light. 10 uL of PCR product was then digested with 4U *Hinf*I and 1X NEB Buffer 2 at 37°C for 12 hrs, followed by an 80°C enzyme deactivation step of 20 mins. Restriction digest products were electrophoresed on a 15 cm 3.5% agarose gel for 120 min at 80 V, which was then poststained in a 0.01% solution of ethidium bromide in 1X TAE buffer for 40 min and visualised under ultraviolet light. *Hinf*I digestion of fragments containing the T allele produced two fragments of 175 bp and 23 bp while fragments containing the C allele remained undigested by *Hinf*I.

The MTHFR A1298C polymorphism was genotyped by PCR followed by high resolution melt (HRM) analysis. The PCR protocol was as follows: 1X PCR buffer, 1.5 mM MgCl_2_, 0.2 mM dNTPs, 0.3uM forward primer, 0.3uM reverse primer, 1.6uM SYTO9, 1U GoTaq, and 40 ng of DNA. The primer sequences were obtained from a previous study [26] and were validated using an RFLP approach to genotype positive controls as described previously [27]. The PCR followed by high resolution melting analysis was conducted on a Qiagen Rotor-Q (Qiagen, Doncaster, VIC, Australia) and the thermocycling conditions were as follows: 95°C for 5 mins, then 95°C for 5 seconds and 60°C for 10 seconds for 45 cycles. PCR products were melted from 78°C to 88°C at 0.1°C increments every 2 seconds. Amplicon melting temperature (Tm) occurred at 83°C and three separate melt curves were obtained corresponding to the three genotypes AA, AC, and CC.

### 2.4. MTRR Genotyping

The MTRR A66G polymorphism was genotyped by PCR followed by HRM analysis. The PCR protocol was as follows: 1X PCR buffer, 1.5 mM MgCl_2_, 0.2 mM dNTPs, 0.3 uM forward primer, 0.3 uM reverse primer, 1.6 uM SYTO9, 1U GoTaq. The primer sequences were obtained from a previous study [28] and were validated using an RFLP approach described previously [27]. The PCR followed by HRM analysis was conducted on a Qiagen Rotor-Q and the thermocycling conditions were as follows: 95°C for 5 mins, then 95°C for 5 seconds and 60°C for 10 seconds for 45 cycles. PCR products were melted from 75°C to 85°C at 0.1°C increments every 2 seconds. Amplicon Tm occurred at 80°C and three separate melt curves were obtained corresponding to the three genotypes AA, AG, and GG.

### 2.5. MTHFD1 Genotyping

The MTHFD1 G1958A polymorphism was genotyped by PCR followed by HRM analysis. The PCR protocol was as follows: 1X PCR buffer, 1.5 mM MgCl_2_, 0.2 mM dNTPs, 0.3 uM forward primer, 0.3 uM reverse primer, 1.6 uM SYTO9, 1 U GoTaq. The primer sequences were obtained from a previous study [29] and were validated using an RFLP approach to genotype positive controls as described previously [29]. The PCR followed by HRM analysis was conducted on a Qiagen Rotor-Q and the thermocycling conditions were as follows: 95°C for 5 mins, then 95°C for 5 seconds and 60°C for 10 seconds for 45 cycles. PCR products were melted from 79°C to 89°C at 0.1°C increments every 2 seconds. Amplicon Tm occurred at 84°C and three separate melt curves were obtained corresponding to the three genotypes AA, AG, and GG.

### 2.6. Statistical Analysis

Power analysis for this study was performed using the Power for Genetic Analyses software [30]. Genotype counts were tabulated for each of the four markers and genotype and allele frequencies were computed for each marker. All groups were tested for and found to be within Hardy-Weinberg equilibrium (HWE). Genotype and allele frequencies were compared between case and control groups for each marker using the chi-square test, with two and one degrees of freedom, respectively. All statistical analyses were performed using Microsoft Excel 2010 for Windows (v14.0).

### 2.7. Interaction Analysis

Given the possibility that each variant may only contribute a small independent effect which may not be detectable as statistically significant in our case control cohort, we also performed interaction analysis using the MDR 2.0 software version beta 8.4. The MDR program was designed to test for interactive genetic effects on a trait even if the independent effects are nonsignificant [22].

In the MDR software, main effect (one-locus) models, two-locus models, or N-locus models are generated, and each model is assessed for prediction accuracy by dividing the dataset into multiple sets, with one set excluded from model-training and then used to test the model. The process of division, model-training, and model-testing is repeated multiple times to cross-validate each model. Testing accuracy (TA) and cross-validation consistency (CVC) are then used to evaluate the overall best model. Permutation testing can then be performed on the dataset using an additional module called MDRpt, which evaluates the significance of the model TA [22].

Before performing the MDR analysis, all markers were examined for correlation using PLINK's pairwise LD function [31], to identify SNPs that may be collinear. None of the four markers were found to be significantly correlated (*r* ^2^ > 0.85) and all were used in the MDR analysis. Missing genotypes were then imputed by mode substitution. Software default settings were used except that the cross-validation was repeated 100 times, and paired analysis was selected. The model with the highest TA and CVC was determined to be the best model and significance *P* values were then generated using 10,000 permutations in the MDR permutation testing module (MDRpt) version 1.0 beta 2.

## 3. Results

This study has more than 90% power to detect a relative risk of at least 1.5 for all markers. Genotype and allele frequencies for all four markers are shown in Table 2. Of the 409 cases and 409 controls, 377 cases (92.2%) and 393 controls (96.1%) and 368 cases (90.0%) and 386 controls (94.4%) were successfully genotyped for the MTHFR C677T and MTHFR A1298C markers, respectively. For the MTRR A66G marker, 360 cases (88.0%) and 358 controls (87.5%) were successfully genotyped, and for the MTHFD1 marker, 364 cases (89.0%) and 360 controls (88.0%) were successfully genotyped. Samples which exhibited ambiguous melt curves for high resolution melt analysis were not counted resulting in a lower genotyping success rate compared to the RLFP assay. Both case and control groups across all four markers were found to be in HWE (*P* > 0.05).

For MTHFR, there was no statistically significant difference between the genotype frequencies of cases and controls for either the C677T marker (*χ* ^2^ = 0.03, *P* = 0.99) or the A1298C marker (*χ* ^2^ = 1.10, *P* = 0.58). There was also no statistically significant difference between the allele frequencies of cases and controls for either the C677T (*χ* ^2^ = 0.02, *P* = 0.88) or the A1298C (*χ* ^2^ = 0.16, *P* = 0.69) polymorphisms. For the C677T marker, there was no observed trend in either the genotype or allele frequencies, with the TT genotype frequency at 8.7% for cases and 8.9% for controls, and the T allele frequency at 31.8% for cases and 32.2% for controls. For the A1298C marker, there was an increased AA genotype frequency in cases (44.8%) compared to controls (42.0%), though this trend was less apparent in A allele frequency in cases (65.4%) compared to controls (64.4%). The observed minor allele frequencies in the control group for both the C677T marker (T allele, 32.2%) and the A1298C marker (C allele, 35.6%) conformed well with expected control frequencies for each marker (C677T, T allele, 31%; A1298C, C allele, 36%) as determined in the Hap-Map CEU population (Utah residents of Northern European ancestry).

Similarly, for the MTRR A66G polymorphism, there was no statistically significant difference between either the genotype frequencies of cases and controls (*χ* ^2^ = 0.92, *P* = 0.63), or the allelic frequencies of cases and controls (*χ* ^2^ = 0.79, *P* = 0.37). The GG genotype frequency was 18.1% for cases and 20.7% for controls, while allele frequencies showed a trend of decreased G allele frequency in cases (44.7%) compared to controls (47.1%). Although the genotype frequencies of our control group seemed markedly different to the Hap-Map CEU frequencies with 52.8% of heterozygotes in our control population compared to only 34.0% in the Hap-Map CEU population, the allelic frequencies of our control group (A allele, 52.9%) and the Hap-Map CEU population (A allele, 55.0%) were similar.

For the MTHFD1 G1958A polymorphism, there was no statistically significant difference between cases and controls for either the genotype frequencies (*χ* ^2^ = 1.73, *P* = 0.42) or the allelic frequencies of cases and controls (*χ* ^2^ = 0.31, *P* = 0.58). The GG genotype frequency was 32.7% for cases and 28.9% for controls, while the G allele frequency was 55.8% for cases and 54.3% for controls. The observed allele frequencies for our control group (G allele, 54.3%) was similar to expected allele frequencies as determined by the Hap-Map CEU population (G allele, 58.0%). Case and control genotype frequencies were also analysed by gender (Table 3). There were 436 females (218 cases and controls) and 382 males (191 cases and controls); all groups were found to be in HWE. No significant differences between cases and controls were detected when analysed by gender and therefore all further analyses were performed using the entire population.

**Figure 2:**
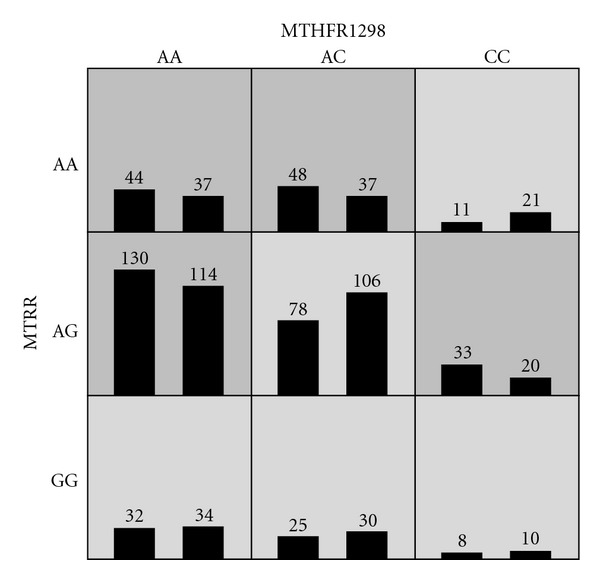
Frequencies of cases and controls for the best MDR model (MTHFR1298-MTRR). Low-risk combined genotypes are indicated by light grey cells and high-risk combined genotypes are indicated by dark grey cells.

For the MDR analysis, the best MDR models for the one SNP (main effect), two SNP, and three SNP combinations are shown in Table 4. The best model had a TA of 0.5526 and CVC of 100/100, and was a two-SNP model containing the MTHFR1298 and MTRR markers.  Figure 2 shows the frequency of cases and controls for each multilocus genotype in the model. The light grey cells indicate genotype combinations (MTHFR1298-MTRR) of the low risk group and the dark grey cells indicate genotype combinations of the high risk group. When multidimensional data under the MTHFR1298-MTRR model were collapsed into one dimension (risk level), the frequency of controls was higher in the low-risk group compared to cases (201 controls, 154 cases) while case frequency was higher in the high-risk group compared to controls (255 cases, 208 controls). There appears to be a moderate synergistic effect between MTHFR1298 and MTRR and a weaker synergistic effect between MTHFR677 and MTHFD1. However, the best model (MTHFR1298-MTRR model) was found not to be significantly associated with case status (*P* = 0.2367).

## 4. Discussion

We investigated the homocysteine pathway variants MTHFR C677T, MTHFR A1298C, MTRR A66G, and MTHFD1 G1598A in an Australian Caucasian population for association with EH. There was no statistical difference between our case and control groups for either genotype or allele frequencies for any of the markers studied, indicating no detected association between these four markers and EH in our case-control population. However, given the sample size limitation, we could not rule out the possibility that these variants contributed a modest effect on EH in this cohort (OR < 1.5) that was not detectable as statistically significant in this study, therefore, we conducted the interaction analysis using an MDR approach. We found that the best model indicated an interaction between the two SNPs MTHFR A1298C and MTRR A66G, which was found to be nonsignificant by permutation testing. This may reflect the fact that the mechanism by which hHcy can cause hypertension is not well understood. However, a recent study in human umbilical artery smooth muscle cells reported an increase in the proliferation of vascular smooth muscle cells through the Hcy-mediated differential regulation of cyclin A and D1, which led to an increase in intima media thickness [32]. Another study on mesenteric arteries in mice showed that hHcy decreased bioavailability of nitric oxide by decreasing the expression of endothelial nitric oxide synthase through the activation of matrix metalloproteinases during oxidative stress [33]. These studies seem to implicate hHcy in vascular remodelling or vasoconstriction, suggesting a possible mechanism for EH development.

MTHFR has been among the most studied genes in relation to Hcy and folate metabolism, with regard to a variety of diseases ranging from neural tube defects to CVD and EH. Previous studies have shown that the MTHFR variants C677T and A1298C have been associated with both higher levels of Hcy [15] and EH risk [34] directly. Currently, MTHFR C677T has been studied in relation to hypertension in 29 published papers indexed on the PubMed database, 25 of which were included in a meta-analysis conducted in 2007, which concluded that there was an overall association of MTHFR C677T with hypertension, with an OR of 1.343 (95%CI 1.198–1.505) [35]. Overall, this is less than a two fold increase in OR for EH cases, which may indicate that larger sample sizes would be needed to detect a modest effect. However, the sample size for this study (409 cases, 409 controls) is larger than the largest study included in the meta-analysis (247 cases, 249 controls). The meta-analysis also showed high heterogeneity between studies, with only 6 published studies showing a clear statistically significant association with EH, while 19 published studies had a nonsignificant OR [35]. However, studies included were from various countries and ethnicities, suggesting that population differences in allele frequency and association may have been confounded.

Another meta-analysis of Hcy metabolizing enzymes and risk of coronary heart disease consisting of 23 studies reported an association of the C allele of the MTHFR A1298C with myocardial infarction with an OR of 1.37 (95% CI 1.03–1.84) [36]. However, conflicting results were obtained when the controls were subdivided and analysed with the C allele being associated with a decreased risk of coronary heart disease (CHD) in hospital-based case-control studies while it was associated with an increased risk of CHD in population-based case-control studies [36]. Overall, findings for MTHFR have therefore been varied and may represent differing MTHFR allele frequencies between ethnic groups, low power of small studies to detect modest effect sizes on CVD and EH risk, or a true lack of association between MTHFR variants and CVD and EH.

MTRR and MTHFD1 have both been shown to carry variants which decrease enzymatic activity and disrupt either MTR reactivation (for MTRR) or purine synthesis (for MTHFD1) though MTHFD1 has not been previously studied in relation to EH. The MTRR A66G polymorphism has been associated with increased Hcy levels [20]. However, a recent study of the MTRR A66G marker reported a lack of association with both Hcy concentration and risk of vascular disease [37], and a 2002 study in adolescents failed to find an association with EH [38]. This is the first study which has examined both MTRR and MTHFD1 in association with adult EH, and though individually they do not appear significantly associated with EH risk, it is possible that each variant confers only a modest effect. We hypothesised that an interaction analysis may have greater power to detect tiny effect sizes for each marker, and therefore conducted an interaction analysis using MDR. Though synergistic effects were detected, especially between MTHFR A1298C and MTRR, the best model was not found to be significant and therefore these effects may not be due to a true interaction between the variants, or may need to be confirmed in a larger case-control cohort. The interaction analysis did not detect MTHFR C677T as part of the best model, which is unexpected as the strongest individual association has been previously found between this variant and EH [35]; however, this may be because MTHFR C677T is not significantly associated with EH in this population.

Current data from this and other studies suggest that genes within the Hcy pathway are not significantly associated with an increase in EH risk, including the well-studied marker MTHFR C677T. Additionally, given that each gene may confer a modest effect to EH risk, a polygenic profile analysis of genes in the Hcy pathway may be warranted. Additionally, future studies should measure plasma Hcy levels to determine whether a combination of these markers influences Hcy levels overall. Our study could not verify whether Hcy levels are significantly different between our cases and controls, and whether individual markers or combinations of markers influence EH risk through elevating Hcy levels. Further, the effects of diet on Hcy levels and EH risk should be controlled for in any future analysis as protective diet such as high folate intake may abrogate an increased genetic risk to EH due to genetic variations in the Hcy pathway.

## Figures and Tables

**Table 1:** SNP and assay information.

Gene	Location	rs number	SNP	AA change	Assay	Primer sequence	Product size	Enzyme	Digest fragment
MTHFR	1p36.3	rs1801133	C677T	A222V	PCR-RFLP	FWD: 5′ TGAAGGAGAAGGTGTCTGCGGGA 3′	198 bp	*Hinf*I	C-198 bp
						REV: 5′ AGGACGGTGCGGTGAGAGTG 3′			T-175 bp and 23 bp
MTHFR	1p36.3	rs1801131	A1298C	E429A	HRM	FWD: 5′ CTTTGGGGAGCTGAAGGACTACTAC 3′	163 bp	N/A	N/A
						REV: 5′ CACTTTGTGACCATTCCGGTTTG 3′			
MTRR	5p15.31	rs1801394	A66G	I22M	HRM	FWD: 5′ GCAAAGGCCATCGCAGAAGACAT 3′	118 bp	N/A	N/A
						REV: 5′ AAACGGTAAAATCCACTGTAACGGC 3′			
MTHFD1	14q24	rs2236225	G1958A	R653Q	HRM	FWD: 5′ CATTCCAATGTCTGCTCCAA 3′	254 bp	N/A	N/A
						REV: 5′ GTTTCCACAGGGCACTCC 3′			

AA: amino acid; PCR-RFLP: polymerase chain reaction-restriction fragment length polymorphism; HRM: high resolution melt.

**Table 2:** Genotype and allele frequencies.

Marker	Genotype frequencies				Allele frequencies		
MTHFR C677T	CC	CT	TT	Total	C	T	Total

Case	170 (45.1%)	174 (46.2%)	33 (8.7%)	377	514 (68.2%)	240 (31.8%)	754
Control	175 (44.5%)	183 (46.6%)	35 (8.9%)	393	533 (67.8%)	253 (32.2%)	786
Test statistic	*χ* ^2^ = 0.03, *P* = 0.99 (*α* = 0.05)				*χ* ^2^ = 0.02, *P* = 0.88 (*α* = 0.05)		

MTHFR A1298C	AA	AC	CC	Total	A	C	Total

Case	165 (44.8%)	151 (41.0%)	52 (14.2%)	368	481 (65.4%)	255 (34.6%)	736
Control	162 (42.0%)	173 (44.8%)	51 (13.2%)	386	497 (64.4%)	275 (35.6%)	772
Test statistic	*χ* ^2^ = 1.10, *P* = 0.58 (*α* = 0.05)				*χ* ^2^ = 0.16, *P* = 0.69 (*α* = 0.05)		

MTRR A66G	AA	AG	GG	Total	A	G	Total

Case	103 (28.6%)	192 (53.3%)	65 (18.1%)	360	398 (55.3%)	322 (44.7%)	720
Control	95 (26.5%)	189 (52.8%)	74 (20.7%)	358	379 (52.9%)	337 (47.1%)	716
Test statistic	*χ* ^2^ = 0.92, *P* = 0.63 (*α* = 0.05)				*χ* ^2^ = 0.79, *P* = 0.37 (*α* = 0.05)		

MTHFD1 G1958A	GG	AG	AA	Total	G	A	Total

Case	119 (32.7%)	168 (46.2%)	77 (21.1%)	364	406 (55.8%)	322 (44.2%)	728
Control	104 (28.9%)	183 (50.8%)	73 (20.3%)	360	391 (54.3%)	329 (45.7%)	720
Test statistic	*χ* ^2^ = 1.73, *P* = 0.42 (*α* = 0.05)				*χ* ^2^ = 0.31, *P* = 0.58 (*α* = 0.05)		

**Table 3:** Genotype and allele frequencies, analysed by gender.

Marker		Genotype frequencies				Allele frequencies		
MTHFR C677T		CC	CT	TT	Total	C	T	Total

Male	Case	76 (46.1%)	75 (45.5%)	14 (8.5%)	165	227 (68.8%)	103 (31.2%)	330
	Control	73 (39.9%)	93 (50.8%)	17 (9.3%)	183	239 (65.3%)	127 (34.7%)	366
Test statistic		*χ* ^2^ = 1.35, *P* = 0.51 (*α* = 0.05)				*χ* ^2^ = 0.95, *P* = 0.33 (*α* = 0.05)		
Female	Case	94 (44.3%)	99 (46.7%)	19 (9%)	212	287 (67.7%)	137 (32.3%)	424
	Control	102 (48.6%)	90 (42.9%)	18 (8.6%)	210	294 (70%)	126 (30%)	420
Test statistic		*χ* ^2^ = 0.77, *P* = 0.68 (*α* = 0.05)				*χ* ^2^ = 0.53, *P* = 0.47 (*α* = 0.05)		

MTHFR A1298C		AA	AC	CC	Total	A	C	Total

Male	Case	77 (45.8%)	68 (40.5%)	23 (13.7%)	168	222 (66.1%)	114 (33.9%)	336
	Control	86 (47.5%)	75 (41.4%)	20 (11%)	181	247 (68.2%)	115 (31.8%)	362
Test statistic		*χ* ^2^ = 0.57, *P* = 0.75 (*α* = 0.05)				*χ* ^2^ = 0.37, *P* = 0.54 (*α* = 0.05)		
Female	Case	88 (44%)	83 (41.5%)	29 (14.5%)	200	259 (64.8%)	141 (35.3%)	400
	Control	76 (37.1%)	98 (47.8%)	31 (15.1%)	205	250 (61%)	160 (39%)	410
Test statistic		*χ* ^2^ = 2.13, *P* = 0.35 (*α* = 0.05)				*χ* ^2^ = 1.24, *P* = 0.27 (*α* = 0.05)		

MTRR A66G		AA	AG	GG	Total	A	G	Total

Male	Case	45 (28.1%)	81 (50.6%)	34 (21.3%)	160	171 (53.4%)	149 (46.6%)	320
	Control	40 (24.2%)	92 (55.8%)	33 (20%)	165	172 (52.1%)	158 (47.9%)	330
Test statistic		*χ* ^ 2^ = 0.93, *P* = 0.63 (*α* = 0.05)				*χ* ^2^ = 0.11, *P* = 0.74 (*α* = 0.05)		
Female	Case	58 (29%)	111 (55.5%)	31 (15.5%)	200	227 (56.8%)	173 (43.3%)	400
	Control	55 (28.5%)	97 (50.3%)	41 (21.2%)	193	207 (53.6%)	179 (46.4%)	386
Test statistic		*χ* ^2^ = 2.29, *P* = 0.32 (*α* = 0.05)				*χ* ^2^ = 0.77, *P* = 0.38 (*α* = 0.05)		

MTHFD1 G1958A		GG	AG	AA	Total	G	A	Total

Male	Case	59 (36.9%)	67 (41.9%)	34 (21.3%)	160	185 (57.8%)	135 (42.2%)	320
	Control	48 (28.4%)	89 (52.7%)	32 (18.9%)	169	185 (54.7%)	153 (45.3%)	338
Test statistic		*χ* ^2^ = 4.05, *P* = 0.13 (*α* = 0.05)				*χ* ^2^ = 0.63, *P* = 0.43 (*α* = 0.05)		
Female	Case	60 (29.4%)	101 (49.5%)	43 (21.1%)	204	221 (54.2%)	187 (45.8%)	408
	Control	56 (29.3%)	94 (49.2%)	41 (21.5%)	191	206 (53.9%)	176 (46.1%)	382
Test statistic		*χ* ^2^ = 0.01, *P* = 0.99 (*α* = 0.05)				*χ* ^2^ = 0.00, *P* = 0.95 (*α* = 0.05)		

**Table 4:** Best MDR models.

Model	Training accuracy	Testing accuracy	CV consistency	*P* value
MTHFR1298	0.5270	0.4951	97/100	0.9621
MTHFR1298_MTRR	0.5575	0.5526	100/100	0.2367
MTHFR677_MTHFR1298_MTRR	0.5681	0.4780	68/100	0.9863

CV: cross-validation.
